# Study Protocol: The Coaching Alternative Parenting Strategies (CAPS) Study of Parent-Child Interaction Therapy in Child Welfare Families

**DOI:** 10.3389/fpsyt.2020.00839

**Published:** 2020-09-02

**Authors:** Akhila K. Nekkanti, Rose Jeffries, Carolyn M. Scholtes, Lisa Shimomaeda, Kathleen DeBow, Jessica Norman Wells, Emma R. Lyons, Ryan J. Giuliano, Felicia J. Gutierrez, Kyndl X. Woodlee, Beverly W. Funderburk, Elizabeth A. Skowron

**Affiliations:** ^1^ Center for Translational Neuroscience, University of Oregon, Eugene, OR, United States; ^2^ Department of Counseling Psychology and Human Services, University of Oregon, Eugene, OR, United States; ^3^ Department of Psychology, University of Oregon, Eugene, OR, United States; ^4^ Department of Psychology, University of Washington, Seattle, WA, United States; ^5^ Center for Excellence, Graduate School of Medicine, University of Tennessee, Knoxville, TN, United States; ^6^ Department of Psychology, University of Manitoba, Winnipeg, MB, Canada; ^7^ Department of Developmental & Behavioral Pediatrics, University of Oklahoma Health Sciences Center, Oklahoma City, OK, United States

**Keywords:** Parent-Child Interaction Therapy, child maltreatment, parenting, self-regulation, socio-cognitive processes, cardiac physiology, high density electroencephalogram, parent-child interaction

## Abstract

**Background:**

Child maltreatment (CM) constitutes a serious public health problem in the United States with parents implicated in a majority of physical abuse and neglect cases. Parent-Child Interaction Therapy (PCIT) is an intensive intervention for CM families that uses innovative “bug-in-ear” coaching to improve parenting and child outcomes, and reduce CM recidivism; however, the mechanisms underlying its effects are little understood. The Coaching Alternative Parenting Strategies (CAPS) study aims to clarify the behavioral, neural, and physiological mechanisms of action in PCIT that support positive changes in parenting, improve parent and child self-regulation and social perceptions, and reduce CM in child welfare-involved families.

**Methods:**

The CAPS study includes 204 child welfare-involved parent-child dyads recruited from Oregon Department of Human Services to participate in a randomized controlled trial of PCIT versus a services-as-usual control condition (clinicaltrials.gov, NCT02684903). Children ages 3–8 years at study entry and their parents complete a pre-treatment assessment prior to randomization and a post-treatment assessment 9–12 months post study entry. Dyads randomized to PCIT complete an additional, abbreviated assessment at mid-treatment. Each assessment includes individual and joint measures of parents’ and children’s cardiac physiology at rest, during experimental tasks, and in recovery; observational coding of parent-child interactions; and individual electroencephalogram (EEG) sessions including attentional and cognitive control tasks. In addition, parents and children complete an emotion regulation task and parents report on their own and their child’s adverse childhood experiences and socio-cognitive processes, while children complete a cognitive screen and a behavioral measure of inhibitory control. Parents and children also provide anthropometric measures of allostatic load and 4–5 whole blood spots to assess inflammation and immune markers. CM recidivism is assessed for all study families at 6-month follow-up. Post-treatment and follow-up assessments are currently underway.

**Discussion:**

Knowledge gained from this study will clarify PCIT effects on neurobehavioral target mechanisms of change in predicting CM risk reduction, positive, responsive parenting, and children’s outcomes. This knowledge can help to guide efforts to tailor and adapt PCIT to vary in dosage and cost on the basis of individual differences in CM-risk factors.

## Introduction

CM is a serious public health problem in the United States, affecting nearly one million children each year, with serious negative developmental outcomes including trauma, dysregulation, and a host of behavioral challenges that are resistant to intervention (1–4). CM is difficult to treat, and CM recidivism is difficult to prevent in families once it occurs (5). Why? CM parents often lack positive parenting skills, with meta-analyses documenting that CM parents are more hostile and controlling, less affectionate, and less likely to engage positively with their children as compared to non-CM parents (6, 7). Psychoeducational approaches (i.e., teaching parents new skills) and traditional family interventions have not proven to be effective for strengthening positive parenting skills and reducing CM risk in families where CM has already occurred (8).

Parent-Child Interaction Therapy (9) (PCIT) is one of few parenting interventions that successfully reduces harsh, aversive parenting and decreases 2.5-year rates of re-abuse from 50% to 19% in CM families, while improving the quality of parent-child relationships and child behavioral adjustment (8, 10) across ethnically diverse CM families with children ages 4 to 12 years old (10, 11) (Chaffin et al., 2004, 2011). PCIT is a behavioral parent-training program grounded in social learning, attachment, and family systems theories. As such, the intervention is designed to improve child functioning by strengthening warm, responsive parenting, interrupting patterns of harsh, coercive interactions, and promoting safe and effective child management skills to halt physical child abuse and neglect (12, 13). PCIT is assessment-driven, in that parents complete ratings of child behavior and therapists observe and code 5 min of parent-child interactions at the outset of each session to inform the session focus. Goals for parenting include: 1) using specific positive parenting skills such as targeted praise (i.e., labeled praise) for prosocial child behaviors, 2) selectively ignoring minor misbehavior, 3) refraining from use of sarcasm or criticism, and 4) implementing consistent, predictable and safe discipline techniques to promote child compliance that can be generalized to a broad range of situations. Parents receive live coaching from a PCIT therapist who provides immediate prompts *via* “bug-in-the-ear” technology while the parent interacts with their child, creating opportunities in the moment for parents to adjust their behavior and immediately correct errors on the spot. This approach allows for parents to practice skills with their child in a safe, constructive environment, and for therapists to intervene immediately to prevent coercive interactions.

**Figure 1:**
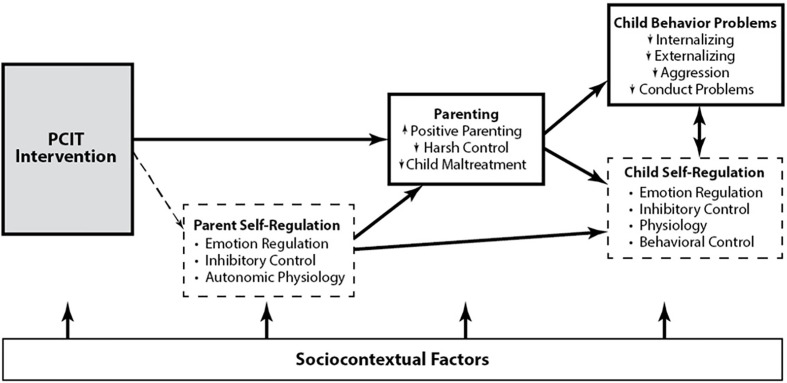
Conceptual model of the study. Known (in bold) and hypothesized (in dashed) PCIT intervention effects on child maltreating (CM) parent and child outcomes.

Though PCIT is effective for CM families, there is limited knowledge about how and for whom it works best. The CAPS study will investigate potential candidate target mechanisms of change, including neural, physiological, and behavioral processes related to parent self-regulation and socio-cognitive processes (see **Figure 1** for conceptual model). Self-regulation skills are important for competent, prosocial parenting. CM parents display deficits in neurobehavioral indices of self-regulation as compared to non-CM parents (14, 15). Parents at high risk for CM display deficits in executive function, greater reactivity to both child-specific and neutral stimuli, difficulties regulating their emotional and behavioral responses, and physiological dysregulation in the form of higher resting heart rate, greater sympathetic activation, and lower resting respiratory sinus arrhythmia (RSA), than do non-maltreating parents (16, 17). New research indicates that harsh parenting is fueled by neurobehavioral markers of dysregulation in CM parents (18, 19) and that PCIT may improve parent-reports of parent and child emotion regulation skills from pre- to post-treatment (20). Yet, no research to date has examined the effects of PCIT on neurobehavioral markers of parent self-regulation in a randomized clinical trial, and whether the effects of PCIT on parenting are mediated by improvements in parent regulation.

CM parents also tend to hold negative, threat-sensitive attributions of their children (21). These negative parental attributions have, in turn, been shown to impact both engagement in and efficacy of parenting interventions (22, 23). Still, no published study to our knowledge has examined how in-the-moment parent coaching (e.g., PCIT) may change parental attributions in welfare-involved parents. PCIT’s active, live-coaching approach during parent-child play sessions provides CM parents with opportunities to reappraise their children’s intentions and behavior in more developmentally informed and prosocial ways. Thus, the CAPS study is designed to test pathways for PCIT’s effects on positive parenting and reduced CM risk through changes in parents’ social cognitions (i.e., softening CM parents’ harsh attributions of their children).

Finally, the CAPS study will test for PCIT-driven positive effects on children’s developing self-regulation skills in addition to the previously well-documented gains achieved in child behavior outcomes. CM-exposed children are doubly challenged by (a) stress overload through exposure to specific acts of CM and (b) heightened environmental and biological risk for poor self-regulation that is inextricably linked to their parents’ own self-regulation difficulties (24). Research has documented the detrimental effects of CM exposure on children’s developing capacities to regulate emotion, cognition, physiology, and behaviour (25, 26), including heightened autonomic responding to interpersonal hostility, dysregulated emotion, enhanced event-related potential (ERP) responses to negative emotion (27, 28), problems with cortisol regulation (29), and impairments in inhibitory control (19, 30). Beyond the broad social address effects of CM on children’s developing self-regulation, the quality of proximal parenting that children receive also has a significant effect on their developing neurobehavioral capacities for self-regulation, with warm, sensitive parenting enhancing self-regulation development, and harsh, aversive parenting further compromising it (31). However, current knowledge about the effects of parenting interventions on the self-regulation skills of children who remain in the custody of their CM parents is limited. Knowledge gained in this clinical trial of PCIT will help to answer key questions about the mechanisms of action in PCIT with child welfare families. In summary, the CAPS study has three specific aims, as follows:

1. Evaluate the effects of PCIT on parenting practices, parents’ socio-cognitive processes (i.e., self-perceptions, attributions of child), and children’s behavioral and health outcomes, relative to a services as usual (SAU) control condition. To do so, we will utilize microsocial codes of parent-child interactions and survey outcomes of parenting attributions and child behavior to characterize change from pre- to post-treatment. The effects of PCIT on CM recidivism will be assessed through Department of Human Services (DHS) Child Welfare chart reviews conducted at study entry and 9–12 months post-treatment.
2. Characterize the effects of PCIT on neurobehavioral markers of parent self-regulation (i.e., cardiac autonomic control, cognitive control of attention, and inhibition) relative to the SAU condition. We will examine direct effects and the extent to which changes in self-regulation mediate the effects of PCIT on parenting behavior and CM recidivism.
3. Characterize the effects of PCIT on neurobehavioral markers of children’s self-regulation (i.e., cardiac autonomic control, cognitive control of attention and inhibition, and behavioral control) relative to children in the SAU condition, and examine the extent to which outcomes are mediated through PCIT effects on parenting.

## Methods

### Participants

A sample of 204 parents and their 3- to 8-year-old children with records of suspected or indicated maltreatment (i.e., physical abuse and/or physical neglect) were recruited from the Oregon DHS. Families with child sexual abuse were excluded from the study because adults with a history of sexually maltreating children are contraindicated for PCIT services. Initially, 228 parent-child dyads were recruited, with 24 dyads withdrawing before completing the pre-treatment assessments. Thus, *N* = 204 parent-child dyads were randomized to condition (PCIT or control). Caregivers primarily include biological or adoptive mothers who live in the home and have a significant custodial role with the child (*n* = 180), and approximately, 12% were father-headed dyads (*n* = 24). Initial recruitment efforts focused on mothers, as many child welfare families are headed by mothers who are single parents, and because our underlying theoretical model centers on the role of neurobehavioral regulatory processes best characterized to date in human and nonhuman primate studies of mother-child dyads. Upon request from DHS collaborators, we extended our recruitment efforts to include fathers as they composed a significant proportion of primary caregivers in child welfare-involved families.

Participating parents identified their race/ethnicity as: European American/White (*n* = 183), Hispanic American/Latina (*n* = 20), African American/Black (*n* = 10), Asian/Asian American (*n* = 2), Pacific Islander (*n* = 6), and Native American/Alaskan Aleut (*n* = 24). Participating children were identified as: European American/White (*n* = 190) Hispanic American/Latina (*n* = 31), African American/Black (*n* = 18), Asian/Asian American (*n* = 6), Pacific Islander (*n* = 7), and Native American/Alaskan Aleut (*n* = 40). Parents who identified themselves or their children as bi- or multi-racial were asked to select all races that applied, resulting in more responses than participants. Roughly, 1% of caregivers had less than a 7^th^ grade education (*n* = 3), 2% completed junior high (8^th^ grade; *n* = 4), 13% completed some high school (9th – 12^th^ grade; *n* = 27), 50% had a high school or equivalent education (*n* = 101), 14% completed a technical or vocational certificate (*n* = 29), 13% completed an associate’s degree, (*n* = 27), 5% completed a bachelor’s degree (*n* = 11), and 1% completed a graduate degree (*n* = 2). At pre-treatment assessment, 53% of caregivers were unemployed (*n* = 109), 6% held temporary or seasonal jobs (*n* = 13), and 40% held stable jobs (*n* = 81). According to the U.S. Department of Health and Human Services 2020 federal poverty guidelines based on gross monthly income and family size, 77% of all participant households would be characterized as living below the poverty line.

### Screening Procedures

This randomized controlled trial received ethics approval from the University of Oregon Institutional Review Board as well as the State of Oregon’s Department of Health and Human Services. A DHHS certificate of confidentiality was obtained to further protect the identity of research subjects in the study. Parents and guardians provided written informed consent to participate, and children gave verbal assent prior to engaging in any study procedures.

In partnership with Oregon DHS Child Welfare and Self Sufficiency divisions, eligible families were identified and recruited to participate in the clinical trial. DHS staff members identified eligible families from their database if the primary caregiver had 1) no history of perpetrating sexual abuse and 2) a child between 3 and 8 years old. Following this pre-selection process within DHS, a core member of the CAPS research team contacted each family to invite them to participate in the study and further screen them for eligibility. Families were free to participate or decline, and were informed that they would be randomized to either the PCIT intervention or would be provided services as usual after completion of their second visit. Interested families were screened for eligibility on the following criteria: (a) the participating parent was at least 18+ years old at study entry and (b) is the participating child’s biological or custodial parent; (c) the participating child was between 3 and 7 years old at study entry; (d) no parent or caregiver in the home was a documented child sexual abuse perpetrator per child welfare records, (e) the parent spoke sufficient English to engage in the assessment, and (f) the parent provided written informed consent to participate.

### Clinical Trial Design

The CAPS study (clinicaltrials.gov, NCT02684903) is being conducted over the course of approximately 4 years and includes three total assessments with families. Two assessments were completed for all study families (pre-treatment and post-treatment), and a mid-treatment assessment was completed for PCIT families only. Each of the pre- and post-treatment assessments consisted of two separate visits that were scheduled approximately one week apart. Following completion of both visits in the pre-treatment assessment, families were randomly assigned to one of two conditions in this parallel groups design: PCIT treatment or Family Services-as-Usual (SAU) control. Families were overallocated to the intervention condition at a rate of 1.5:1 to ensure an ample number of families accessed the intervention. Random assignment to condition was retained through the overallocation process (32). Allocation was concealed from research assistants who collect data at all assessment waves.

**Figure 2:**
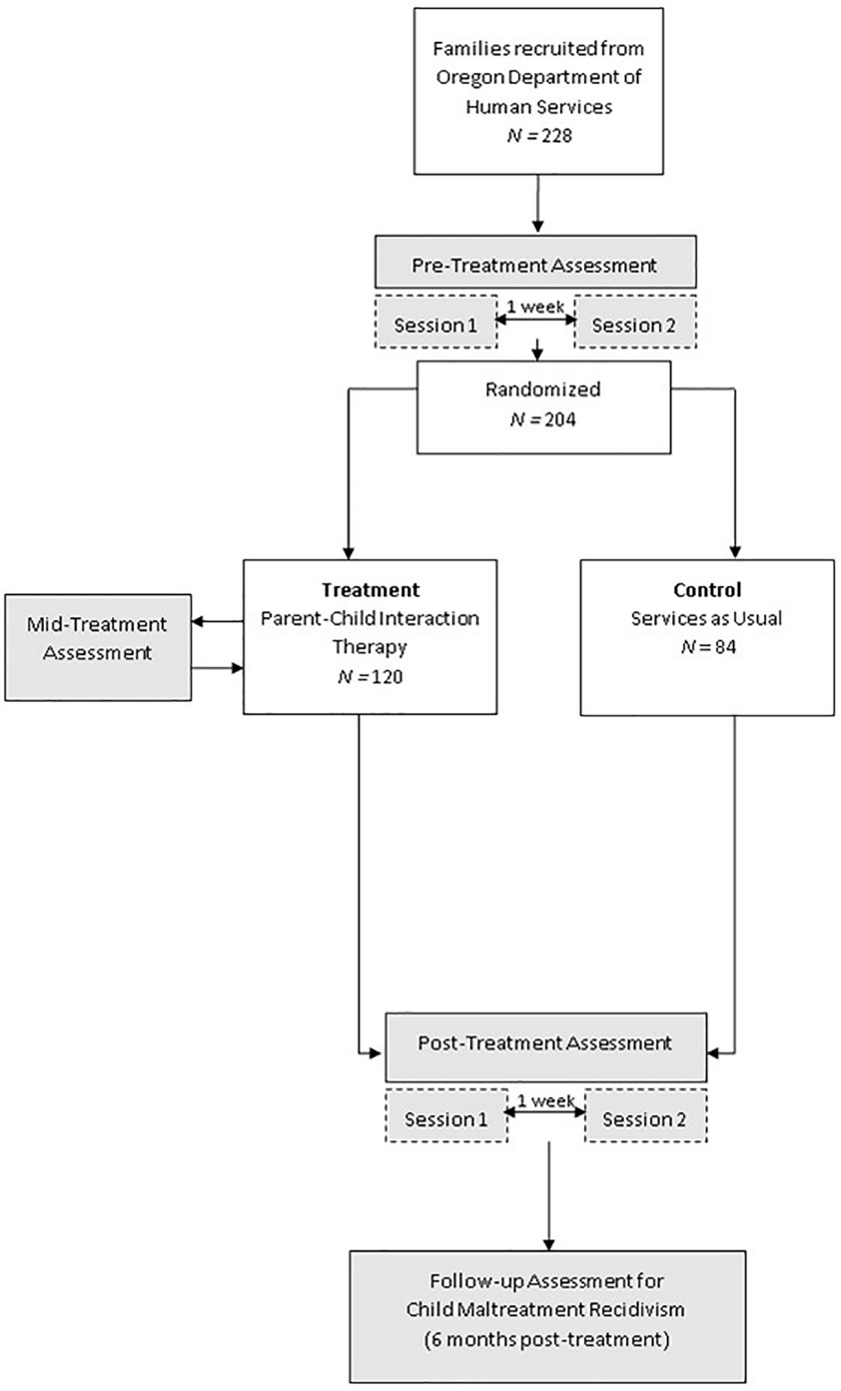
Coaching Alternative Parenting Strategies (CAPS) Study Flowchart.

Pre-treatment assessments were conducted on enrolling families from spring, 2016 through spring, 2019. Mid-treatment and post-treatment assessments are currently ongoing. Families randomized to PCIT complete post-treatment immediately after their last PCIT session, or approximately 9–12 months after study entry for those who discontinued PCIT prematurely. Families randomized to SAU control complete post-treatment approximately 9–12 months after study entry. Post-treatment assessments for the SAU control families are case-control matched with the PCIT group on time from study entry to post-treatment assessments. **Figure 2** shows the CAPS study flow chart.

### Assessment Procedures

Pre-treatment and post-treatment assessments are conducted with all participants in the study, each completed in two successive laboratory visits scheduled one week apart. Mid-treatment assessments are conducted only with PCIT intervention group families, after completion of the first PCIT phase (i.e., child-directed interaction, CDI) and before beginning the second PCIT phase (i.e., parent-directed interaction, PDI). See *PCIT Intervention and Delivery* below for a detailed description of the intervention. Mid-treatment assessments are completed in one laboratory visit. Pre-treatment, mid-treatment, and post-treatment assessments each include joint parent-child tasks, individual child tasks, and individual parent tasks. Cardiac physiology is monitored at rest, with participants watching a neutral video either jointly before parent-child tasks or individually before solo tasks. Cardiac physiology is also measured during all experimental tasks and during recovery periods (where participants again watch a neutral video) immediately following each task (see physiology acquisition details in *Outcome* *Measures* below). For ease of understanding, pre-treatment and post-treatment procedures will be detailed first, and mid-treatment procedures for intervention families will be listed after. Parents are compensated for their time at each visit, are offered paid taxi services or reimbursed for transportation, and children are given a small prize. Detected child maltreatment outcomes are evaluated using the state-wide child welfare administrative database, with matches based on unique identifiers for the participating child and individual unique identifiers for the participating parent. All database matches will be manually checked to confirm any positive matches for future maltreatment reports where the participating study parent is identified as the perpetrator. Any reports made by study therapists will be considered “surveillance effect” reports and treated appropriately in data analyses.

#### Pre- and Post-Treatment Assessment

Following voluntary informed consent procedures, parent-child dyads undergo anthropometric measures (i.e., standing height, weight, and waist circumference) and are fitted with seven disposable, pre-gelled electrodes for the recording of electrocardiogram (ECG) and impedance cardiogram (ICG). ECG is measured from three electrodes placed in a modified Lead II arrangement on the distal right clavicle, lower left rib, and lower right abdomen. ICG is measured from two electrodes placed on the participant’s midline along the top end of the sternum between the two clavicles, at the bottom end of the sternum where the ribs meet, and two electrodes along the spine. ECG and ICG data are wirelessly transmitted *via* an ambulatory impedance cardiograph (Mindware Mobile #50-2303-00; Mindware Technologies, Westerville, OH, USA) to a desktop computer equipped with Mindware’s Biolab (2.4) acquisition software that integrates simultaneously recorded audio and video. Both parents and children wear a vest throughout each visit that secures the wireless ambulatory monitor close to their body and allows them to move freely during the tasks. Cardiac physiology is monitored throughout all tasks at each visit for both parents and children. After children and parents are fitted with the electrodes, a 3-min resting baseline measure of parent and child’s concurrent cardiac physiology is obtained while they are sitting quietly together and watching a neutral video of oceanic animals. Immediately afterward, systolic and diastolic blood pressure and resting pulse are assessed *via* blood pressure cuffs, while the parent and child remain sitting.

Following assessment of initial resting cardiac physiology, dyads complete two joint interaction tasks that are video-recorded and during which cardiac physiology is collected. First, the standard PCIT Dyadic Assessment Protocol (33) employs a standard set and arrangement of toys that are spread out on the playroom floor for all dyads. Parents are provided with an earbud and walkie-talkie to allow for assessors to provide task instructions while parents are alone in the playroom with their child. The PCIT dyadic assessment protocol consists of three 5-min parent-child interactions: a 5-min Child-Led Play task (i.e., *please follow your child’s lead…*); followed by a 5-min Parent-Led Play task (i.e., *now you decide on what you two will play…*), and a 5- min Clean-Up task (i.e., *it’s time for your child to clean up all of the toys*). After the interaction, the parent’s earbud is removed and cardiac physiology is assessed during a 2-min joint recovery before the dyad begins the second interaction task.

**Figure 3:**
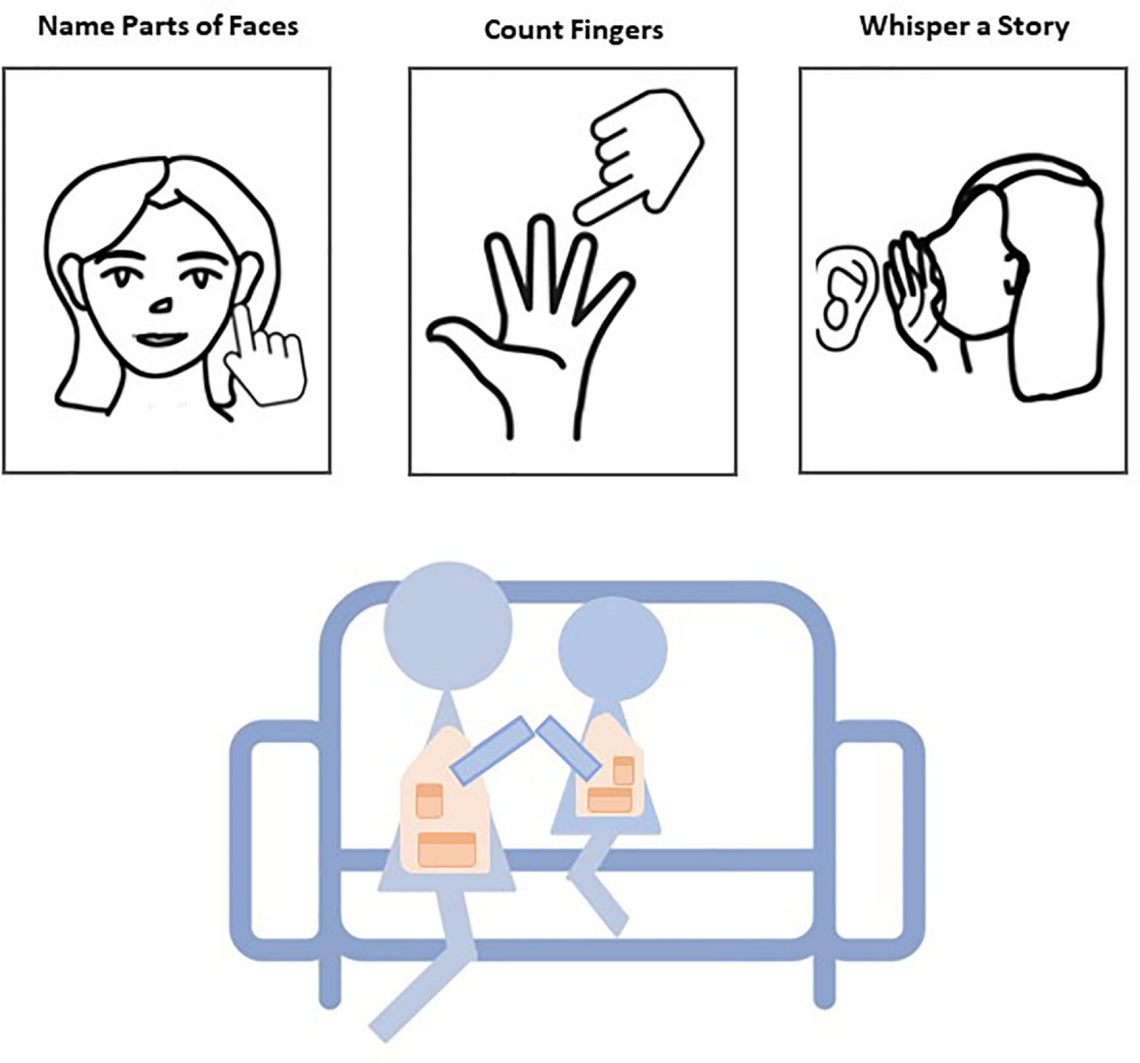
Schematic of the Social Engagement Task is presented. Children and parents engage in three fixed-interval activities that are presented on a screen while cardiac physiology is monitored.

Dyads then complete the Social Engagement task (34) (SET) during which the child and adult are seated in close physical proximity while completing three reciprocal activities: (1) gently pointing to the other’s facial features (i.e., hair, chin, nose, and ears); (2) counting each other’s fingers; and (3) whispering a story in each other’s ear (see **Figure 3**). Children complete the task twice: once with their parent and once with a female research assistant. Each activity is presented for a fixed time interval and the story told by the research assistant is the same with all families. Activity order remains consistent across dyads, whereas the interactive partner condition (i.e., parent or research assistant) is counterbalanced within assessment wave. That is, children are randomly assigned to a partner order that remains constant throughout subsequent assessment waves. Cardiac physiology is assessed in another 2-min joint recovery immediately after completing the SET, and then, blood pressure readings are obtained. Parents and children are given a short break and small snack prior to transitioning into individual tasks.

Parents are taken to a separate testing room in the lab, where the Mindware Mobile carrier is removed and replaced with Biopac carriers for ECG and ICG [Dual Wireless Respiration and ECG BioNomadix Transmitter (BN-Tx); Biopac Systems, Inc.]. Next, parents are fitted with a 256-channel high density Electrical Geodesics Inc. (EGI Philips; Eugene, OR) Hydrocel Geodesic Sensor Net while their child can observe, with the idea that children will be going through a similar process at the following visit. EEG is recorded at a sampling rate of 500 Hz using the EGI net and Net Amp 300 amplifier integrated with Net Station software version 5.2.0.2 (EGI Philips; Eugene, OR). Parents complete a 4-min resting EEG task in which they first close their eyes for 2 min and then fixate on a blank screen for 2 min while seated in a dark room. Next, a 3-min resting baseline measure of cardiac physiology is collected while parents watch a neutral ocean video. Parents complete the executive function tasks [Auditory Attention (AUDAT) and Stop Signal; specified in *Outcome Measures* below] while simultaneous EEG and cardiac physiology are recorded. Parents complete an Emotional Go/No-Go task to conclude visit #1. Meanwhile, children complete two subtests of the Woodcock Johnson-III Tests of Achievement and two brief self-regulatory tasks [Head-Toes-Knees-Shoulders (HTKS); Working Memory] while cardiac physiology is monitored during each task and for 2-min recovery periods after each task. Note that children’s cardiac physiology is recorded during an additional 2-min standing baseline prior to the HTKS task.

Families returned to the lab for a second 2-h visit within one week of their initial visit. During visit #2, children are fitted with a 64-channel EGI Hydrocel Geodesic Sensor with identical EEG equipment and acquisition software to that used with parents. Children complete executive functioning tasks (AUDAT and Zoo Go/No-Go) while simultaneous EEG and cardiac physiology are recorded. Children then complete the Emotional Go/No-Go task while cardiac physiology is recorded. During this time, parents completed questionnaires that allow for the assessment of socio-demographic characteristics, environmental risk, and child behavior (see *Outcome* *Measures*). After parents and children have both completed individual tasks, peak expiratory flow (i.e., cardiovascular function) is measured using a spirometer, and the highest of three values is recorded within-person. Next, whole blood spots are collected from both parents and children to assess intervention effects on metabolic and immune markers. Four to five spots of blood (~50 μl each) are collected on Whatman strips, then are dried, processed, and frozen at −20°C, before samples are transferred for storage in a pad-locked −80°C freezer to undergo enzyme-linked immunosorbent assays (ELISA). All research assistants responsible for collecting blood spots first complete a comprehensive Bloodborne Pathogens training that outlines emergency procedures, safe handling, contact risks, and exposure control plan, prior to working with participants; each research assistant is offered an optional hepatitis-b vaccination at the end of the training before working with study participants. Research assistants use disposable masks that cover the nose and mouth, goggles, and disposable gloves. We also recommend that participating individuals (parent or child) run their hands under warm water to increase blood flow, and remain seated during blood-spot collection to minimize risk of fainting.

At the end of the pre-treatment assessment, the parent is given a sealed, double-blind randomization letter after completing questionnaires and prior to collection of allostatic load measures. If the family is randomized to the PCIT treatment condition, a research assistant reviews the basic structure and goals of PCIT with the parent, gives a brief tour of the PCIT clinical rooms, and schedules the family for an intake session with an available therapist.

#### Mid-Treatment Assessment

Only families who were randomized to the PCIT condition and engaged in treatment sessions are invited to complete a mid-treatment assessment. Written informed consent from the parent and verbal assent from the child are obtained. During this single-visit assessment, dyads complete assessment tasks noted above using identical procedures unless specified here. Parent and child provide anthropometric measures (i.e., height, weight, and waist circumference) and then are fitted with seven disposable, pre-gelled electrodes for recording cardiac physiology during a 3-min joint resting baseline.

Dyads complete the PCIT Dyadic Assessment Protocol during which cardiac physiology data are collected. Cardiac physiology is recorded during a 2-min recovery. Children then complete the Social Engagement task with their parent only (not a research assistant) during the mid-treatment assessment. Cardiac physiology is collected again during a 2-min post-task recovery. Parents and children take a short snack break and then transition to individual tasks.

Parents then complete the Stop Signal task, during which only behavioral data is collected (i.e., no EEG or cardiac physiology) due to time constraints. Parents then complete a subset of the questionnaires that are presented at pre-treatment (see **Table 1**). Separately, children complete a 3-min measure of resting cardiac physiology while standing, followed by the HTKS task. Children’s cardiac physiology is then recorded during a 2-min recovery. Next, the child completes a sitting 3-min resting baseline, followed by the Zoo Go/No-Go task. Neither children nor parents provide EEG data during the mid-treatment assessment due to time constraints. After completion of individual tasks, whole blood spots and measures of peak expiratory flow are collected from both parents and children.

**Table 1:** Survey measures across T1 pre-treatment, T2 mid-treatment, and T3 post-treatment.

Target	Questionnaire	T1	T2	T3
**Child**				
	Adverse Childhood Experiences Scale (35) (ACES)	X		X
	^a^Trauma Symptom Checklist-Young Children (36) (TSCYC)	X		X
	Eyberg Child Behavior Inventory (37) (ECBI)	X	X	X
	Child Eating Behavior Questionnaire (38) (CEBQ)	X		X
	Brief Rating Inventory of Executive Function - Parent Rating of Child (39, 40) (BRIEF or BRIEF-P)	X		X
**Parent**				
	Adverse Childhood Experiences Scale (35) (ACES)	X		
	^b^Brief Symptom Inventory (41) (BSI)	X	X	X
	Behavior Rating Inventory of Executive Function – Adult (42) (BRIEF-A)	X		X
	Child Abuse Potential Inventory (43) (CAPI)	X	X	X
	Parent Attribution Test (44) (PAT)	X	X	X
	Parenting Stress Index (45) (PSI)	X	X	X
	Readiness for Parenting Change (46) (REDI)	X		
	Confusion, Hubbub, and Order Scale (47) (CHAOS)	X	X	X
	Conflict Tactics Scale-2 (48) (CTS-2)	X		X
	Conflict Tactics Scale for Parent and Child (49) (CTS-PC)	X		X
	Work/School Abuse Scale (50) (WSAS)	X		X
	Edinburgh Handedness Inventory (51) (EHI)	X		
	Structural Analysis of Social Behavior (52) (SASB)	X		X
	^c^Interpersonal Mindfulness in Parenting (53) (IEM-P)			X
	^c^Mindfulness Attention Awareness Scale (54) (MAAS)			X
	Services-as-Usual Questionnaire			X

All questionnaires are completed in interview format. An X denotes when the questionnaires were administered across T1 pre-treatment, T2 mid-treatment, and T3 post-treatment.

^a^36 questions were selected for the following scales: Anxiety (ANX), Posttraumatic Stress-Intrusion (PTS-I), Posttraumatic Stress-Avoidance (PTS-AV), Posttraumatic Stress-Arousal (PTS-AR), and Posttraumatic Stress-Total (PTS-TOT).

^b^Only anxiety and depression subscale questions were administered.

^c^These scales were added halfway through the study.

### Outcome Measures

#### Dyadic Parent-Child Interactions

Video-recorded parenting behaviors and child responses during the standard PCIT Dyadic Assessment Protocol (i.e., child-led play, parent-led play, and clean-up tasks) at pre-treatment, post-treatment, and mid-treatment are collected, transcribed and then coded using the well-validated Dyadic Parent-Child Interaction Coding System, Fourth Edition (55) (DPICS-IV). For the PCIT intervention group, DPICS-IV coding is also completed on parent-child interactions during Child Directed Interaction (CDI) sessions (i.e., standard, 5-min child-directed play segment) and standard Parent Directed Interaction (PDI) session segments that begin with the 5-min child-directed free play segment (see *PCIT Intervention and Delivery* below). PCIT session coding is conducted using only the session video-recordings (i.e., without transcripts).

Parenting behaviors that are coded include labeled praises, unlabeled praises, behavior descriptions, and reflections; criticisms, direct and indirect commands, questions, and neutral talks. Children’s compliance and non-compliance behaviors in response to parent commands are coded. Commands where children have no opportunity to comply are also coded. Coders complete 20 h of intensive, hands-on training prior to coding study assessments, and continue to meet regularly to maintain 80% inter-rater reliability. All coders are blind to participants’ condition group and assessment wave. Parent and child behaviors are coded sequentially and summed to calculate a task average for each behavior. For assessment visits (i.e., pre-treatment, mid-treatment, and post-treatment), values are summed in 30-sec epochs within each task and summarized into task averages. Reliability coding will be completed on 20% of study families, with criterion set to 80% inter-rater reliability.

#### Cardiac Physiology

Behavioral and procedural event markers are inserted into the physiological data stream at the time of data collection to time-lock behavioral and physiology data within tasks. From ECG/ICG recordings of cardiac physiology, RSA and pre-ejection period (PEP) are assessed as indices of the parasympathetic and sympathetic nervous systems, respectively. RSA is derived from high-frequency heart rate variability measured in the ECG (children, 0.24 to 1.04 Hz; adults, 0.12 to 0.40 Hz). PEP is derived by measuring the distance between the Q-point of the ECG and the B-point of the dZ/dt wave, indexing the time interval between opening of the left ventricle and ejection of blood into the ventricle. Heart rate is evaluated from the ECG as the number of R-R wave intervals per minute. Both RSA and PEP are measured in 30-s epochs, except during the Emotional Go/No-Go task (described below) during which they are assessed for the task duration. All data is cleaned offline using Mindware HRV Analysis software version 3.1.3. Data are visually inspected and cleaned for movement artifacts and equipment errors.

#### Neurophysiology

EEG is acquired from parents and children during rest periods and completion of executive function tasks (i.e., Stop Signal, Go/No-Go, and AUDAT) during T1 pre-treatment and T3 post-treatment sessions only. EEG is recorded at a sampling rate of 500 Hz using an EGI Hydrocel Geodesic Sensor Net integrated with Net Station software (Electrical Geodesics Inc; Eugene, OR). After recordings are completed, raw EEG files are exported from Net Station in simple binary format to prepare them for importing to Matlab for preprocessing using the EEGLAB toolbox. After preprocessing, continuous EEG files are epoched into task-specific bins by time-locking EEG to event codes synchronized with events of interest, to yield ERPs for analysis. Each epoch is subjected to standard artifact rejection procedures, rejecting individual epochs where: (1) ocular electrodes showed electrical changes of 75 microvolts or greater or (2) where electrode activity at the scalp exceeds 200 microvolts; however, these are followed up with more stringent artifact rejection thresholds if artifacts are not sufficiently removed on an individual participant basis. Final processed ERP files for a given participant for each task will consist of all artifact-free epochs of interest, relative to a 200-ms pre-stimulus baseline. Grand averages are created for each task by averaging across groups of participants.

##### Attentional Control

**Figure 4:**
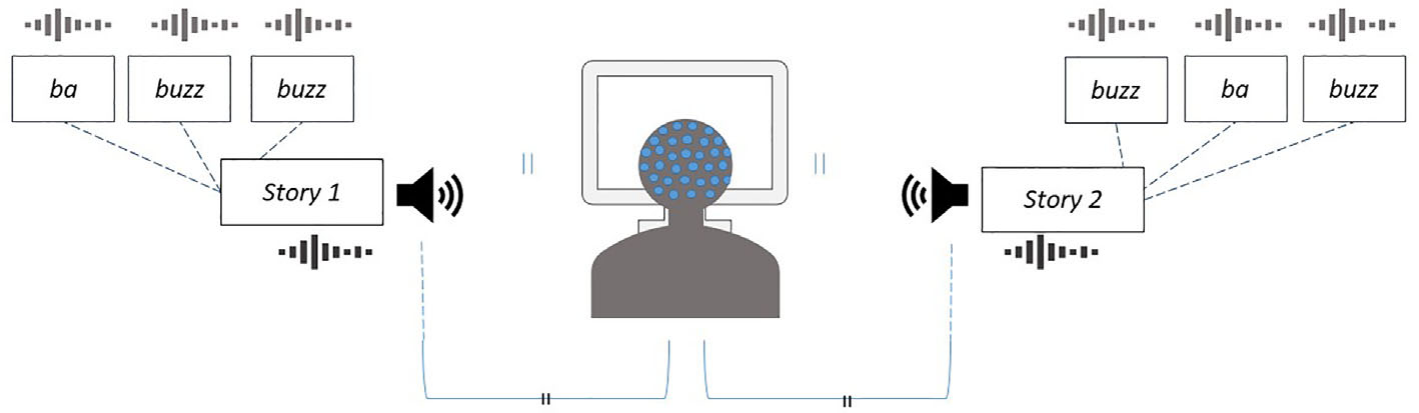
Schematic of the Auditory Attention Task to assess attentional control. While fitted with an EEG net and electrocardiograph, both parents and children are instructed to listen to a children’s story presented from a free-field speaker situated 90° to their right or left, while another story is playing simultaneously from the speaker on the opposite side. During each story, one speaker presents a male voice while the other speaker presents a female voice, each reading different narratives ranging from 2.5 to 3.5 min in length and edited to remove pauses greater than 1 second. An arrow at the bottom of the screen reminds the listener which speaker to attend to. ERPs are recorded according to stimuli (ba, buzz) that are superimposed over each of the stories.

Parents and children individually complete the Auditory Attention Task (56, 57) (AUDAT) to assess attentional control. Participants listen to one of two children’s stories presented simultaneously in separate free-field speakers situated 90° to their left and right sides (see **Figure 4**). During each story, one speaker presents a male voice while the other speaker presents a female voice, each reading different narratives ranging from 2.5 to 3.5 min in length and edited to remove pauses greater than 1 second. An arrow on the screen reminds the participant which story to attend to. Selective attention during the task is assessed *via* ERPs recorded to 100-ms sound probes (i.e., ba, buzz sounds) superimposed on the to-be-attended and unattended stories. Each participant attends to four separate stories over the duration of the task, with direction of attention counterbalanced across gender of narrator (male or female) and side of speaker (left or right) with a pseudo-randomized order. Immediately after each story, the participant is asked three comprehension questions to ensure they were attending to the appropriate narrative.

##### Inhibitory Control

**Figure 5:**
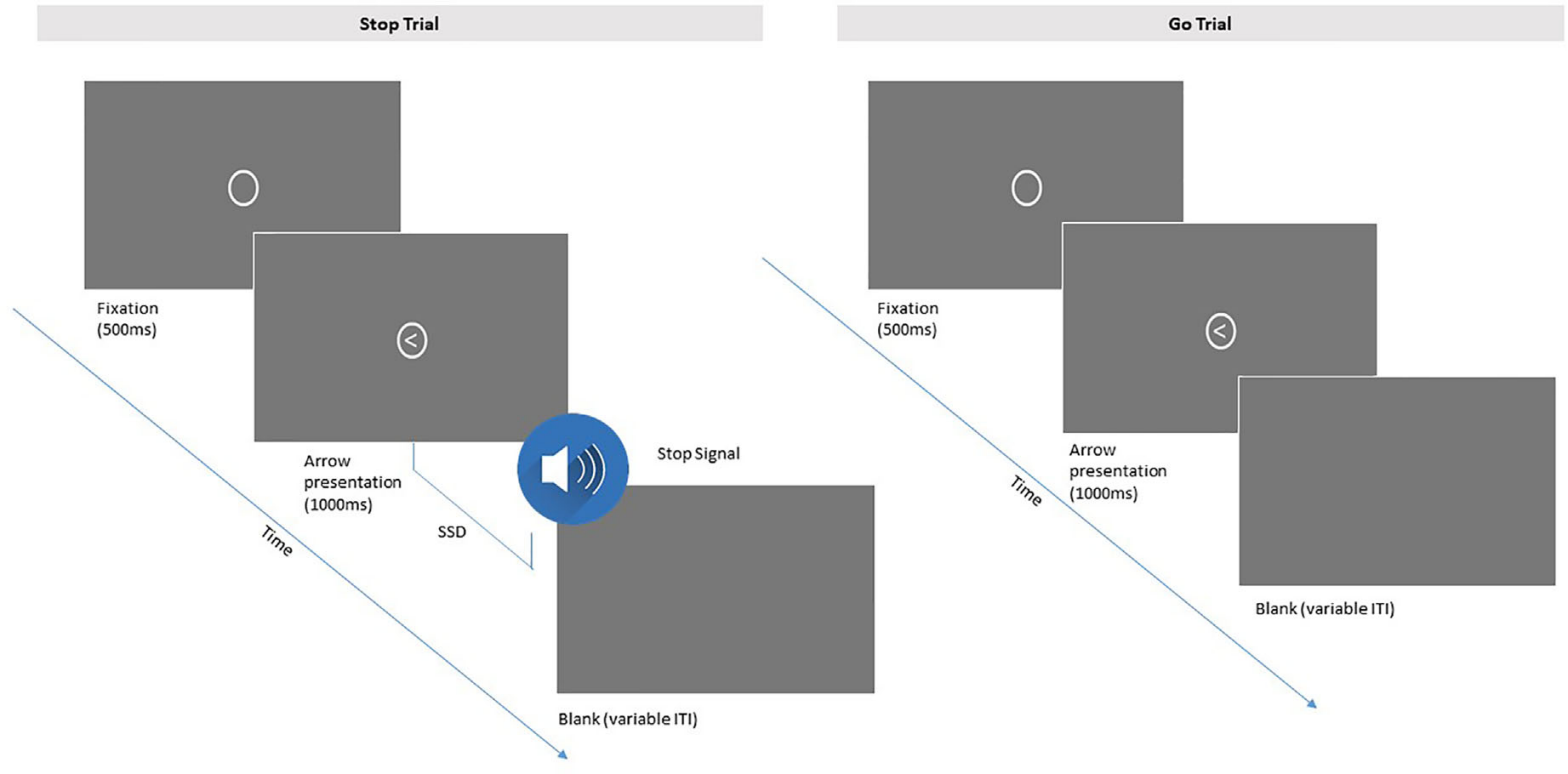
Depiction of the Stop Signal Task sequence for parents. Participants press the arrow on a keyboard that corresponds to the direction of the arrow in each trial. Participants are instructed to withhold a response when an auditory stop signal is played immediately after the go signal at a variable latency (SSD, stop signal delay; ITI, intertrial interval).

Parents complete two 6-min blocks of the Stop Signal task (58) to assess response inhibition and impulse control. As shown in **Figure 5**, each trial consists of a cue indicating the start of a trial (500 ms), followed by an arrow pointing either left or right (at 1:1 relative frequency) that serves as a go signal (1,000 ms) and then an inter-trial interval of variable duration. Parents are instructed to press the left or right arrow as quickly as possible in response to the go signal. On 25% of the trials, an auditory stop is played after the go signal at a variable latency known as the stop-signal delay (SSD), in response to which parents are instructed to withhold their button press. A stop-signal response time (SSRT) is calculated as the difference between the speed of the stop process and the stop signal delay, and reflects efficiency of the inhibitory control process. Each block of the task consists of 128 trials (32 stop trials) and lasts 6 min. Total testing time is approximately 12 min for 256 trials. Of interest are ERPs time-locked to the stop signal and ERPs time-locked to responses, for example, N2 and P3 ERP components shown to be enhanced in amplitude on trials that are correctly inhibited.

**Figure 6:**
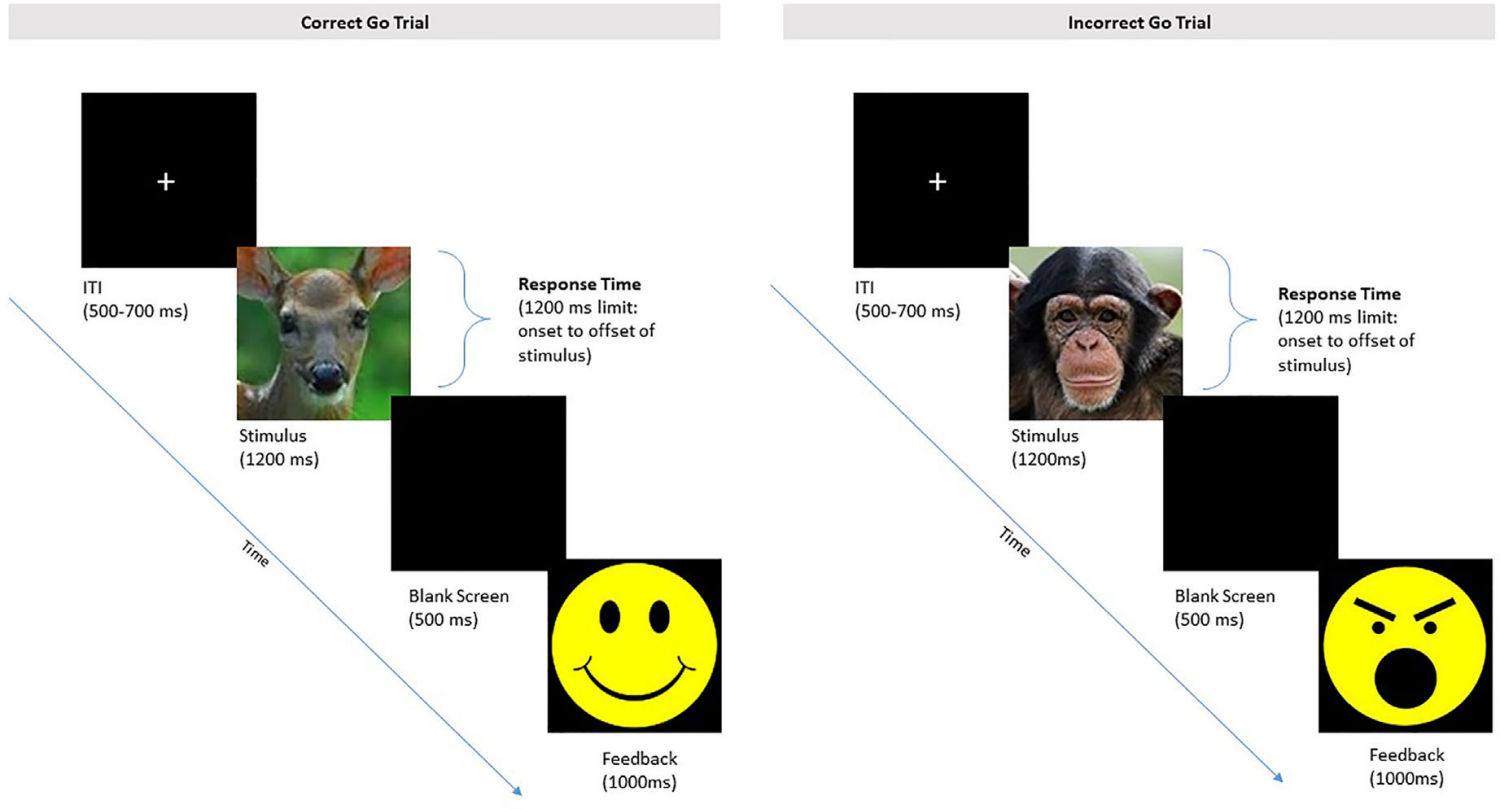
Depiction of the Zoo Go No-Go Task for children. Participants respond with a button-press when an animal (e.g., deer) appears in the go task. Children are instructed to withhold a response when the monkey appears. Children are presented with smiley-face feedback for correctly withholding a response, and angry-face feedback for incorrectly responding to a monkey.

Participating children complete four 2.5-min blocks of a Zoo Go/No-Go task (59) to measure inhibitory control. As shown in **Figure 6**, children are presented with a story of a zookeeper and instructed to respond by pressing on a button box each time a zoo animal appears on the computer monitor (go signal). After 12 practice trials, they are told to withhold their response each time they see a monkey. On No-Go trials (i.e., monkey stimulus), children are given feedback for their responses, with a smiley emoticon for correct responses and correctly withheld responses, and an angry emoticon for incorrect responses (i.e., did not withhold or did not respond). After a brief practice period that includes the monkey and emoticon feedback, children complete four blocks of 45 trials each, with short breaks in between blocks, for a total of 180 trials. Of these, 33% of trials per block are no-go trials.

Children also complete the Head Toes Knees Shoulders task (60) (HTKS), adapted for use with ages 4–8, to assess behavioral response inhibition, and with McClelland’s modifications, is appropriate for use with children aged 3 to 7 years. Children are introduced to four instructions, “touch your head,” “touch your toes,” “touch your shoulders,” and “touch your knees.” After a series of non-conflict trials in which they do as instructed, children are told to respond using the “opposite” rule to the examiner (e.g., touch head when examiner says touch toes). A total of 30 trials are presented, with responses scored as correct, self-corrected, or incorrect. The reliability of the HTKS task is high and the task is predictive of academic achievement (60–62).

##### Emotion Regulation

**Figure 7:**
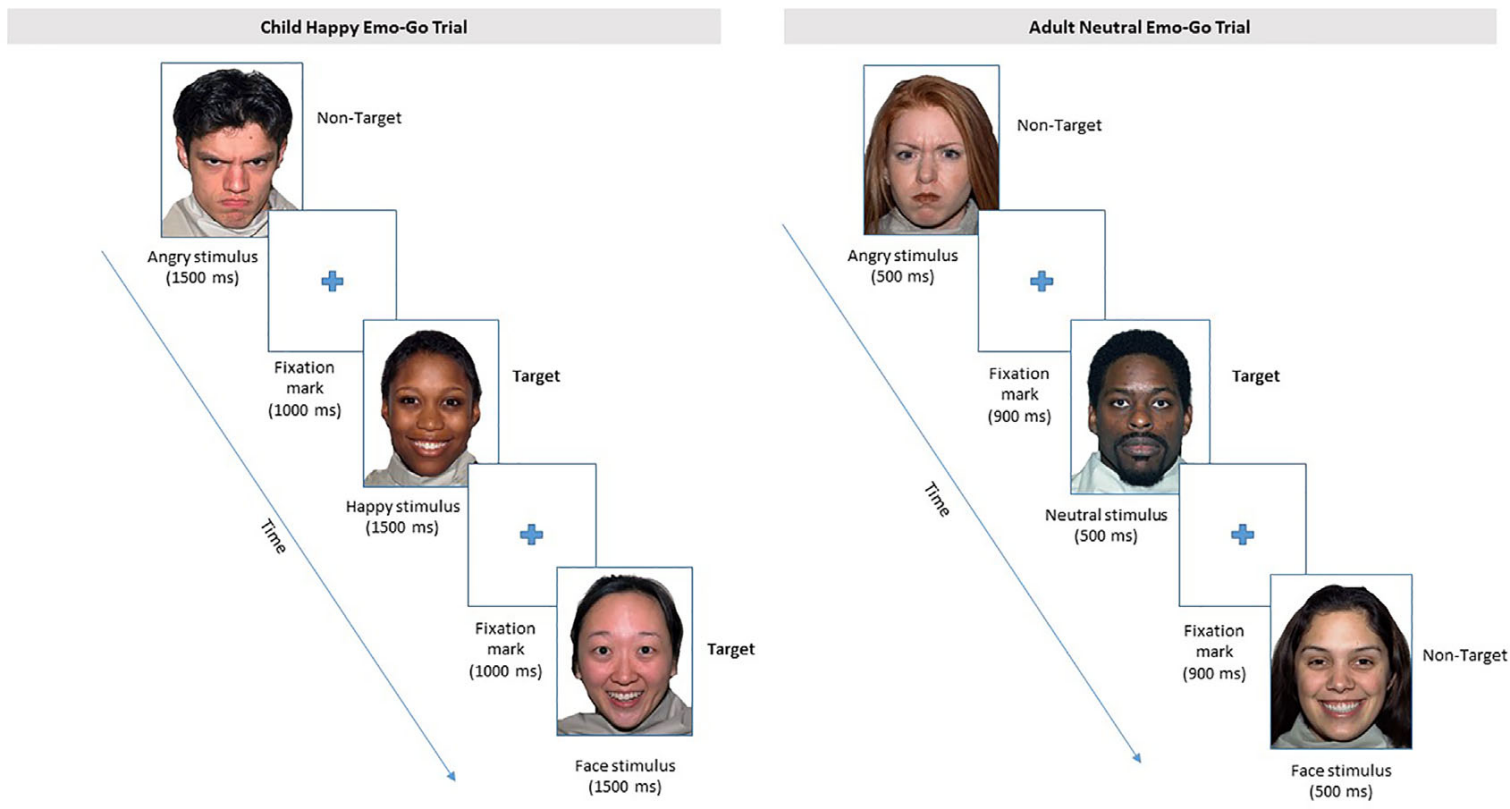
From the Emotional Go/No-Go task, depictions of the “happy” block for children, and of the “neutral” block for parents are presented. Other emotion blocks follow a similar schema for children and adults, respectively. Participants are instructed to respond with a button press to the target emotion of each block and withhold a response for all other emotions in that block. Face stimuli were selected from the MacBrain Face Stimulus set available at www.macbrain.org.

Parents and children also complete an Emotional Go/No-Go task (63, 64) to assess emotion regulation (see **Figure 7**). Stimuli are images of neutral, angry, happy, sad, and fearful facial expressions for parents, and only neutral, happy, or angry for children. Parents and children each complete an age-appropriate version of this task, in which they are instructed to press a response key when a target emotion is presented, and refrain from responding when a distractor emotion is presented. Dependent variables include correct responses to targets, and false alarms to distracters.

Parents complete eight blocks of a five-stimulus paradigm with neutral, angry, happy, sad, or fearful facial expressions. The target designation is counterbalanced across blocks of trials, such that each participant responds to four blocks with emotional targets and four blocks with neutral targets, in random order. Each block consists of 50% emotionally valent faces and 50% neutral faces. In this way, the participant is presented with each emotion twice: one block in which the emotional face is the target, and one block in which the emotional face is the distracter (i.e., the neutral face is the target). Each block consists of 30 trials for a total of 240 trials.

Children complete a version of the Emotional Go/No-Go task consisting of four blocks of a three-stimulus paradigm with neutral, angry, or happy facial expressions. Only happy (50% of trials) and angry (50% of trials) faces are presented for the first two blocks, which consist of 18 trials each. Children are instructed to respond to happy faces in the first block and angry faces in the second block. Neutral faces are introduced in the third and fourth blocks (20 trials each), but instructions remain the same. That is, children are instructed to respond to angry faces in the third block, and to happy faces in the fourth block. Block three presents 20% happy, 40% angry, and 40% neutral faces; and block four presents 40% happy, 20% angry, and 40% neutral faces. Total testing time is 10–15 min.

##### Child Cognitive Screen

Children complete two achievement-based subtests of the Woodcock-Johnson III Tests of Achievement (65) to assess math problem solving skills (Test 10: Applied Problems) and language development (Test 14: Picture Vocabulary). Next, children complete a brief, computerized Working Memory span task (66) in which they are instructed to replicate progressively larger sets of images (e.g., ball, cup, and bow). Children must press 3D representations of objects on a touch screen in sequence. Two trials are presented at each span length until the child completes all trials correctly or completes two consecutive incorrect trials at a given span length.

##### Survey Measures

Parents are asked to report on both their own and their child’s adverse life experiences, attributions, socio-emotional functioning, general health, and relationships. In addition, parents report on their parenting stress, children’s trauma symptoms and children’s behavior. All questionnaires are completed in interview format with a trained research assistant. Parents are given a small booklet of scales for Likert-type questions, which they can point to when responding. Responses are entered into Qualtrics by the research assistant and automatically scored. **Table 1** includes a list of survey measures.

### PCIT Intervention and Delivery

PCIT is an intensive, behavioral parent-training intervention model grounded in social learning, attachment, and family systems theories that uses live skills coaching of parent-child interactions. PCIT for CM families is comprised of three sequential modules. Each session is scheduled at the convenience of the family and availability of the therapist with limited constraints on the time of day. In other words, families were scheduled for PCIT sessions in the morning, afternoon, and evening on weekdays and on weekends per family preferences.

#### Motivational Enhancement

The first module consists of a clinical intake plus two motivational enhancement (ME) sessions drawn from Chaffin and colleagues’ six-session group-based ME module (10), adapted for delivery in an individual session format, totaling 2.5 h. ME sessions are thought to be important for two primary reasons: 1) child welfare-involved parents may be unmotivated initially to change parenting behavior; and 2) PCIT requires parent activity in session (active skills practice to overlearned criteria) and homework assignments, and it cannot be consumed passively (10). Activities in the ME sessions include parents weighing the pros and cons of their current parenting strategies, reviewing a taped testimonial from volunteer program graduates, and completing a decisional balance exercise to encourage development of self-efficacy motivations, beliefs, and expectations (10).

#### PCIT: Child-Directed Interaction

CDI is delivered *via* one didactic “CDI teach” session with parents, followed by live-coached parent-child dyadic sessions, in which parents are coached to use a set of “PRIDE” skills (praise, reflection, imitation, description, enjoyment) during “special time” play, ignore minor child misbehavior, and avoid criticism, sarcasm, and other negative behaviors. During the coaching sessions, parents wear a small earpiece while the therapist coaches *via* a headset from the other side of a one-way mirror, providing positive feedback, support, and guidance. Each session is 50–60 min long and consists of: a 5-min check-in and review of homework, 5-min of DPICS coding to inform coaching goals for that session, 20 to 30 min of in-the-moment coaching, and 10 min to assign homework and check-in with the parent once again.

#### PCIT: Parent-Directed Interaction

PDI is delivered *via* one didactic “PDI teach” session with parents, followed by lived-coached dyadic sessions in which parents are coached to give effective commands and instructions. Parents learn to use contingent praise for child compliance or a brief time-out procedure for noncompliance; PCIT with CM parents eliminates options for use of physical discipline. The PCIT intervention is assessment-driven and includes weekly DPICS coding of parent skills mastery at the beginning of each PCIT session to guide the coaching focus, and rating child behavior problems using the Eyberg Child Behavior Inventory (37) (ECBI). PDI sessions are 50–60 min long with select sessions including brief DPICS-based coding of parents’ CDI and PDI skills that informs the focus of therapist coaching. Initial sessions of PDI (1–2) are often scheduled for 1.5 h to allow enough time for the parent to successfully practice and complete the discipline protocol prior to practicing at home (33) (see PCIT International guidelines).

Parents in the intervention condition are invited to complete a second optional informed consent if they wish for their PCIT therapist to provide a routine progress report to their DHS caseworker. No additional study data, aside from the optional progress reports and any mandated maltreatment reporting, is shared with DHS authorities. Families are provided light snacks at each visit and reimbursed for transportation costs to attend sessions. Treatment is discontinued if the child was removed from the home entirely during the course of treatment; however, families were retained in the study through the T3 post-treatment assessments when possible.

#### Interventionist Training and Fidelity

A team of eight masters or Ph.D. level practitioners completed an intensive 40-h training with Dr. Funderburk and her clinical team of nationally certified PCIT trainers at Oklahoma University Health Sciences Center (OUHSC), and delivered the PCIT intervention to CAPS study families. PCIT therapist training conformed to PCIT International standards for observed case practice and intervention fidelity criteria. Study therapists received ongoing weekly consultation from Master and Level II Trainers at OUHSC, in addition to live, video-based consultation during conduct of their PCIT sessions with study families, and certification after two completed cases. Adherence to the protocols for ME and PCIT sessions are assessed using live, remote, direct observation of sessions and by completing session-by-session fidelity checklists following each session. All PCIT sessions are videotaped to ensure rigorous adherence to the PCIT protocol and a minimum of 10% of videotaped sessions are coded for fidelity by independent observers blind to family outcomes at 90% criterion.

### Services-as-Usual-Control Condition

The Services-as-Usual (SAU) control condition is an ecologically valid, ethical comparison group in which families receive typical services provided by child welfare agencies, including access to a variety of in-home family visitation services, respite childcare, and other individual child counseling and/or parent education training. To characterize the type and dosage of services that all study families may have received, parents complete a Services Utilization Questionnaire *via* interview at post-treatment, indicating how many times in the past 6 months anyone in the family received support from a wide range of social services. Thus, services utilization is tracked *via* parent self-report at T3 post-treatment for all study families.

### PCIT Protocol Modifications

Protocol modifications commonly arise in clinical trials conducted with community samples, with the major concern being a potential dilution of treatment effects on hypothesized outcomes (67, 68). Two adaptations to the initial intervention protocol occurred in this study. *A priori* sensitivity analyses will be conducted in each instance to probe the effects of the intervention protocol modifications. ME Sessions. At launch, the study protocol was designed to allocate PCIT condition families to receive ME sessions only if parents reported low/moderate readiness-to-change (REDI) scores at T1 pre-treatment. This decision was based on findings reported in Chaffin et al.’s (11) clinical trial of PCIT with child welfare families in which participation in ME sessions was associated with reduced treatment effectiveness for parents who were already highly motivated for treatment (i.e., high REDI scores), but beneficial for parents with low/moderate REDI scores. However, approximately 1.5 years into study enrollment, we observed that all CAPS study parents who were randomized to the intervention reported high REDI scores though their drop-out and non-engagement rates paralleled those cited in the parenting intervention literature (69, 70). Therefore, we modified the intervention protocol to deliver ME sessions to all remaining families randomized to the intervention, in line with procedures followed in Chaffin et al. (10, 11). We justified this modification based on two key differences that emerged between samples in the Chaffin et al. (10) clinical trial and this CAPS clinical trial of PCIT with child welfare families. First, Chaffin et al.’s families were mandated to treatment, whereas the CAPS study families were not mandated to participate. Second, Chaffin et al.’s families posted a wide range of high, moderate, and low REDI scores, whereas the CAPS study families were reporting consistently high REDI scores at pre-treatment. Standard PCIT Dosage. The PCIT intervention protocol was initially set at 16–18 total sessions delivered weekly. However, a small number of intervention families enrolled in the early months of the study were given extensions to help them try to achieve PCIT mastery criteria per PCIT International guidelines. Recognizing resource limitations that would prohibit delivery of unlimited sessions to families throughout the course of the clinical trial, at approximately 3 months into the study, we finalized a longer standard PCIT dosage that allowed for a total of N = 22 sessions per family (i.e., 2 ME sessions; 9 total CDI sessions consisting of 1 CDI teach session, followed by up to 8 CDI coaching sessions; and 11 total PDI sessions, consisting of 1 PDI teach session, followed by up to 10 PDI sessions). A total of four intervention condition families received greater than the 22 total sessions (i.e., at *n* = 23, 26, 27, 30 sessions). Further, no family treated prior to implementation of the standard PCIT dosage protocol was denied fewer than 22 PCIT sessions.

## Data Analysis

First, intent-to-treat (ITT) analysis will provide a stringent assessment of PCIT efficacy. Repeated-measures ANCOVAs will take into account variability between and within subjects and enable the detection of group differences by reducing the effects of high individual variability in the sample. We will specify latent models (LGM) with three time points (pre-treatment, mid-treatment, post-treatment), comparing linear and possible nonlinear trajectories for mediating and focal outcomes in the PCIT group. Although several related approaches to ITT analysis for LGM are available, including multigroup analyses and parallel slope processes, we will primarily specify treatment assignment as a binary covariate to evaluate tests of mediated pathways hypothesized in Aim 2. Thus, for example, we will use an LGM approach with rate of change in parent self-regulation and parenting behavior regressed on the ITT contrast, allowing for tests of indirect mediating pathways from treatment to intermediate parent variables (i.e., parent self-regulation) to focal outcomes (i.e., parenting). Following recommendations for indirect effects, we will estimate bootstrapped standard errors for the asymptotic multiplicative indirect terms as well as bias-corrected confidence intervals. We also plan to identify the biobehavioral markers of self-regulation that best forecast risk for early treatment drop-out.

We will also employ Complier Average Causal Effect (CACE) analyses to estimate the effects of intervention for those who comply with treatment at varying degrees, as well as would-be compliers in the control group (71). CACE models, implemented as latent growth mixture models, will use estimated categorical profiles with compliance status included as a training variable to estimate class membership. Utilizing known compliance status in the treatment group and missing values for compliance in the control group will provide an unbiased estimate of how PCIT works by estimating what would happen to the control group, had they been offered the treatment (72, 73).

Given the high comorbidity of physical abuse and neglect, pure subtypes are not as frequent in the CM population. Nonetheless, CM subtype moderation of intervention effects will be examined because important neurobiological and behavioral differences have been observed (74–76). Focusing on simultaneously assessed parent RSA, PEP, ERPs, and behavior taken at pre-, mid-, and post-treatment, we will explore targeted CM group differences in mediational pathways to test whether mediation between treatment and outcome is dependent on CM group characteristics (77, 78). Greater intervention gains are hypothesized for physically abusive parents.

Within-treatment trajectories of session-to-session change in parenting behavior over time will be estimated and those changes mapped onto growth in parent self-regulation. The LGMs will first identify the nature and pattern of change trajectories for both positive and negative parental responses by allowing for linear and polynomial models (79). Parents’ behavioral response percentages will be set as dependent variables for each PCIT session, with session number and response type nested within participants. Once change trajectories are isolated, piecewise growth models that use a segmented coding scheme (80) may be used to test empirically grounded hypotheses for critical intervention change points.

*A priori* sensitivity analyses will be conducted to probe the effects of intervention non-compliance and protocol modifications. Thus, in addition to planned ITT analyses and CACE complier modeling, *a priori* sensitivity analyses will be conducted and results reported, highlighting significance levels and direction of effects.

We used prior results and R 3.5.0 (81), powerMediation (82) and pwr (83) (v1.2-2) packages to estimate power for tests of PCIT main effects *via* ITT analyses and tests of change in parent self-regulation scores as a mediator of PCIT effects on focal parent and child outcomes. For ITT analyses, estimated power was found to be greater than.80 to detect small-to-moderate main intervention effects (Cohen’s *d* = .4). For the mediational models, using the product of standardized beta coefficients comprising the indirect effects (i.e., treatment condition to parenting and parenting to outcome) based on Cohen’s standards, *b* = .01 (small), *b* = .09 (medium), and *b* = .25 (large;.5 ×.5), thus, power is greater than.80 to detect small-to-moderate mediational effects (*b* = .04).

## Discussion

The CAPS study clinical trial is designed to test critically important causal processes regarding PCIT treatment-related changes in CM families. The project addresses current gaps in the CM intervention literature by using a constellation of neurobiological and behavioral measures to test the efficacy of PCIT for strengthening parent emotion regulation and inhibitory control, social perceptions, reducing aversive parenting and CM risk, and supporting improvements in child self-regulation and behavioral outcomes. Findings are expected to inform how PCIT for child welfare families can be optimized, dosed, and sustained.

The CAPS study design has several strengths. First, by using an experimental intervention design, this study will be among the first to clarify the ways in which evidence-based parenting interventions for CM families may affect major neurobiological mechanisms of self-regulation, social perception, and parenting outcomes. Little published research so far has examined the extent of plasticity in such systems within an experimental context. Second, utilization of multimethod assessments (neural, physiological, behavioral, and self-report) allows for reliable construct measurement in parents and children as well as the opportunity to replicate and extend previous studies showing that neural and physiological indices have unique predictive utility. Third, the study uses intensive repeated measures of parenting behavior over time before, during, and after participation in an evidence-based parenting intervention, and links them to laboratory assessments of functioning.

Findings drawn from the CAPS study may hold promise for optimizing CM interventions by extending the focus beyond behavior change alone to (a) determine the sensitivity of CM parent and child neurobiological systems to an evidence-based parenting intervention and (b) identify individual differences that mediate and moderate response to intervention and long-term maintenance of gains.

## Ethics Statement

The protocol for this randomized controlled trial, and all relevant consent forms, recruitment materials, and outcome measures have received ethics approval from the University of Oregon Institutional Review Board as well as the State of Oregon Department of Health and Human Services (DHHS). A DHHS certificate of confidentiality was obtained to further protect the identity of research subjects in the study.

## Author Contributions

ES, AN, RJ, and CS drafted the manuscript. ES conceptualized the study and wrote the grant funding it. ES, LS, KD, CS, BF, and RG developed the study protocol including stimulus/paradigm development and manuals. LS, RJ, FG, CS, KD, JNW, EL, AN, and KW collected data. AN, ES, RJ, CS, LS, RG, EL, KD, FG, JNW, and BF revised sections of the manuscript and/or prepared tables and figures. All authors contributed to the article and approved the submitted version.

## Funding

This research is supported by the National Institute on Drug Abuse of the National Institutes of Health under award number R01DA036533 (ES; Principal Investigator) and the National Center for Advancing Translational Sciences, National Institutes of Health, through award number TL1TR002371 (AN; Pre-Doctoral Fellowship). The content of this manuscript is solely the responsibility of the authors and does not necessarily represent the official views of the National Institutes of Health.

## Conflict of Interest

The authors declare that the research was conducted in the absence of any commercial or financial relationships that could be construed as a potential conflict of interest.
